# Heterogeneous subpopulations in *Escherichia coli* strains acquire adaptive resistance to imipenem treatment through rapid transcriptional regulation

**DOI:** 10.3389/fcimb.2025.1563316

**Published:** 2025-05-30

**Authors:** YunTao Luo, Rong Xu, Bo Yuan, WeiHua Yang, YunHeng Zhou, Yuan Tian, QingZhong Wang

**Affiliations:** ^1^ Clinical Microbiology Laboratory, Shanghai Center for Clinical Laboratory, Shanghai, China; ^2^ Outpatient Department of Clinical Laboratory, Shanghai Tenth People’s Hospital, Shanghai, China; ^3^ Department of Clinical Laboratory, Zhabei Central Hospital of Jing’an District, Shanghai, China; ^4^ Shanghai Institute of Immunity and Infection, Chinese Academy of Sciences, Shanghai, China

**Keywords:** heteroresistance, transcriptional regulation, *Escherichia coli*, non-genetic mechanism, fitness cost

## Abstract

**Introduction:**

Heteroresistance is a well-known phenomenon contributing to treatment failure in bacterial infections. Previous research has traditionally linked it to genetic mechanisms, emphasizing fixed subpopulations with specific resistance mutations. Recent studies appreciated that bacterial subpopulations may not be fixed and independent, but rather dynamically changing. Heteroresistance mechanisms are likely more intricate than mere genetic predisposition alone.

**Methods:**

Our study investigated the role of non-genetically encoded mechanisms in early stages of occurrence and development of heteroresistance through transcriptome analysis and molecular biology experiments.

**Results:**

We identified a clinical *Escherichia coli* strain that, despite no prior antibiotic treatment, still exhibited imipenem heteroresistance. We found that these heteroresistance populations can rapidly acquire adaptive capability for imipenem-resistance through an active and dynamic gene regulatory process. At their highly resistant stage, the transcriptome is primarily characterized by enhanced expression of related genes in exopolysaccharide and peptidoglycan biosynthesis (wcaE, wcaF, mrcB, murA, etc), leading to critical alterations in bacterial intracellular and intercellular structure, including maintaining the integrity of the outer cell membrane and the promotion of biofilm formation. Conversely, in antibiotics-free conditions, these highly imipenem-resistant subpopulations can revert to an imipenem-sensitive state, accompanied by reversed gene expression. Additionally, we discovered that extremely low-level antibiotic exposure can regenerate heteroresistance populations, accompanied by similar pattern of gene expression.

**Discussion:**

Overall, our study revealed non-genetic mechanisms that enable bacterial strains to acquire adaptive imipenem-resistance rapidly. Moreover, preventing hospital-acquired infections should focus not only on eliminating residual bacteria but also on removing residual antibiotics in clinical settings.

## 1 Introduction

In recent years, bacterial population recurrence in antibiotic-sensitive strains during antibiotic treatment is a quite common phenomenon in clinical settings (Huemer et al., 2020). Previous studies have shown that antibiotic resistance is not often in a black-and-white phenotype (Dewachter et al., 2019). Bacterial populations that are classified as susceptible may still harbor subpopulations that survive antibiotic treatment. Heteroresistance is considered a major culprit of surviving subpopulations (Brauner et al., 2016; Stojowska-Swędrzyńska et al., 2021; Eisenreich et al., 2022). Distinction from the dominant antibiotic-sensitive population, a small fraction of seemingly isogenic bacteria could survive and exhibit resistance to a particular antibiotic, irrespective of exposure to high bactericidal concentrations (Andersson et al., 2019; Pan et al., 2023). Clinical data indicate that isolates containing heteroresistant subpopulations are frequently undetected (Band et al., 2021), which can result in inappropriate treatment or therapeutic failure *in vivo* (Howard-Anderson et al., 2021). Furthermore, recent clinical investigations have shown that heterogeneous resistance is often an important stage in the development of antibiotic resistance (Bollenbach et al., 2021). However, in general, heteroresistance, as a mechanism of antibiotic resistance, is not yet fully understood in terms of its origins, development, and recurrence.

Previous studies have mainly supposed that heteroresistance may be attributed to genetically encoded resistance. Some papers have indeed confirmed special genetic mutations in heteroresistant subpopulations (Xu et al., 2020; Kapel et al., 2022; Wang et al., 2022; Doijad et al., 2023). Nevertheless, other studies have also hinted that heteroresistant subpopulations that acquire mutations conferring antibiotic resistance often incur a fitness cost (Lin et al., 2018; Stojowska-Swędrzyńska et al., 2021; Wu et al., 2021; Li et al., 2022). This implies that heteroresistant subpopulations may arise only under strong antibiotic selective pressure. However, in many heteroresistant strains derived from clinical settings, these fitness costs are often mitigated or even absent, suggesting the existence of other alteration mechanisms.

Recent studies have hinted that heteroresistance still displays characteristics of phenotypic plasticity (Eisenreich et al., 2022). Some researchers have found that highly resistant subpopulations are prone to revert to their heteroresistance or even exhibit similar patterns to antibiotic-susceptible bacteria under antibiotic-removed conditions (Wu et al., 2021). Current explanations from genetic mechanisms have mainly attributed to instability of resistant genes (Nicoloff et al., 2019) and compensatory secondary mutations (Durão et al., 2018; Botelho et al., 2019), which lead to the absence of antibiotic resistance or restoration of fitness. However, it is worth noting that these molecular mechanisms only highlight the passive selection of surviving subpopulations during the evolutionary trajectory, which also requires a long-term process (Baquero et al., 2021). In the initial phase, the origins of heteroresistance may represent an active rather than passive manner for bacteria to adapt to the environment by non-genetically encoded means (Bakkeren et al., 2020; Stojowska-Swędrzyńska et al., 2021; Eisenreich et al., 2022; Pan et al., 2023). Unfortunately, so far, many studies have overlooked the potential abilities of strains.

Here, we investigated the transcriptional regulatory mechanism underlying heteroresistance in a clinical *Escherichia coli* strain. The strain without prior antibiotic treatment can also exhibit imipenem (IPM) heteroresistance. Furthermore, heteroresistant populations could rapidly make an adaptive response to environmental alterations through an active gene regulatory process, accompanied by reversal of transcriptional and physiological structure alterations. This kind of non-genetic mechanism allows us to better understand the occurrence and development of heteroresistance and reconsider strategies for eliminating residual bacteria during antibiotic treatment.

## 2 Materials and methods

### 2.1 Strains and growth conditions

The clinical *E. coli* ID1073 strain was isolated from the sputum sample of a diabetic patient in the endocrinology department of a local hospital in the City of Shanghai, China. The isolates were reconfirmed by matrix-assisted laser desorption ionization–time of flight mass spectrometry (Bruker microflex LT, Munich, Germany). ATCC 25922, as a reference strain, was obtained from the Shanghai Center for Clinical Laboratory. For experiment preparation, all bacterial isolates were cultured in Mueller–Hinton broth, Mueller–Hinton agar (Oxoid, Basingstoke, UK), and Luria–Bertani (LB) agar (Sigma-Aldrich, St. Louis, MO, USA) at 35°C.

### 2.2 Antimicrobial susceptibility testing

All isolates were tested for their clinical minimum inhibitory concentration (MIC) and resistance designation in the clinical microbiology lab according to the instructions of the manufacturer (VITEK 2 Compact, bioMérieux, Marcy-l’Étoile, France).

### 2.3 Population analysis profiles

Population analysis profiles (PAPs) were conducted as described previously (Doijad et al., 2023), with some modifications. Briefly, solid agar plates contained 0, 0.06, 0.125, 0.25, 0.5, 1, 2, and 4 μg/mL IPM concentration (Solarbio, Beijing, China). Isolates were grown overnight in Mueller–Hinton broth and were inoculated into agar plates containing different IPM concentrations with serial dilutions (starting inoculum, 1 × 10^8^ cells). Colonies were enumerated after 24 h of growth at 35°C. Heteroresistance was defined as the presence of a subpopulation of cells that can grow at antibiotic concentrations at least eightfold higher than the highest concentration that does not affect the dominant population.

### 2.4 Stability/instability of IPM heteroresistant phenotype

**Figure 1:**
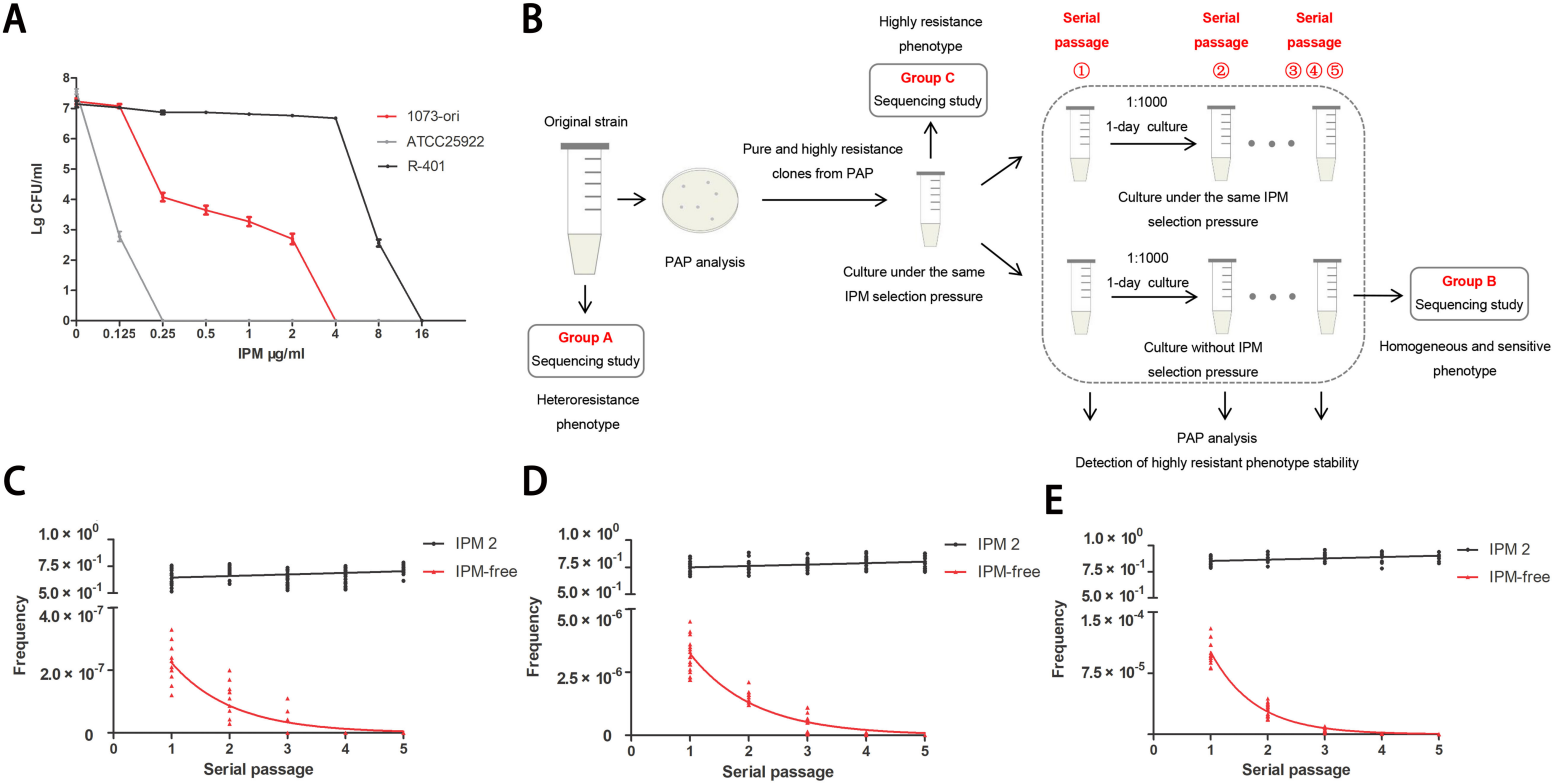
Unstable IPM-heteroresistance subpopulations in a clinical (E) coli strain. **(A)** IPM PAPs of the clinical original ID1073 and reference strains. **(B)** Scheme illustrating that the outline of experimental method to determine the frequency of heteroresistance and the stability/ instability of resistance to IPM among each pure subclone derived from the ID1073 original strain. The 6 clones, capable of growing on plates containing 8×MIC concentrations (IPM: 2μg/mL), were selected from the initial PAP tests. Every clone was inoculated into MH medium with the same IPM selection pressure, growth for 1 day, and then serially passaged with or without IPM selection pressure for parallel experiments. One of the lineages, from parent to derived progeny (group A to C to B, **Table 1**) was selected for sequencing study. **(C-E)** Frequencies of IPM resistant cells among 6 clones in each passage. Each clone was replicated independently three times. IPM2: clones with the same IPM selection (2 µg/ml) as reference; IPM-free: clones without IPM. The X-axis represents the serial passage①-⑤. Each point on the Y-axis represents the frequency of IPM-resistant cells in each clone. Plates from PAP assay with different IPM concentration: **(C)** 8 ×MIC, **(D)** 4×MIC, **(E)** 2×MIC.

The clones identified as highly resistant from the initial PAP tests were selected for further analysis. Selected clones were first cultured overnight in 1 mL of Mueller–Hinton broth with the same IPM concentration (2 µg/mL) as the plate where the clone was initially isolated. Then, each clone was subcultured in parallel under conditions both with and without the presence of antibiotics. In each passage, MICs and the frequency of IPM-resistant cells were determined by Etest and PAP tests (**Figure 1B**). Frequency values were calculated by dividing the number of colonies observed on plates containing antibiotics by the number of colonies on plates without antibiotics.

### 2.5 Growth rate measurement

The starting inoculum was 5 × 10^5^ CFU/well. Bacterial cells were grown in 96-well plates with Mueller–Hinton broth or containing an appropriate IPM dose. Bacterial growth over time was monitored by measuring OD at 600 nm using a microplate reader (Thermo Scientific, Dreieich, Germany). All assays were performed independently in three replicates.

### 2.6 RNA preparation and RNA-Seq analysis

Upon the completion of incubation, the culture supernatant was removed, and bacterial cells were washed three times in sterile Phosphate Buffered Saline (PBS). Total RNA was prepared from different phases of cells in dishes containing TRIzol (1 mL TRIzol per 10 cm^2^; Sigma Chemical Co., St. Louis, MO, USA). RNA extraction and RNA-Seq analysis were performed by Shanghai Majorbio Bio-Pharm Technology Corporation (Shanghai, China). Briefly, after total RNA extraction and DNase I treatment, the mRNA was fragmented into short fragments. Then, cDNA was synthesized using the mRNA fragments as templates. After agarose gel electrophoresis, the suitable fragments were selected for PCR amplification as templates. The templates were then sequenced using the Illumina NovaSeq 6000 sequencer. The data were reported and analyzed on the free online platform of the Majorbio Cloud Platform. A p-value <0.05 and a fold change ≥2 were used as the threshold to judge the significance of the gene expression differences. Furthermore, the DAVID online tool (http://david.ncifcrf.gov) was used to perform Gene Ontology (GO) enrichment analysis and Kyoto Encyclopedia of Genes and Genomes (KEGG) pathway enrichment analysis. p < 0.05 was considered statistically significant (Huang et al., 2007; Huang et al., 2008).

#### 2.6.1 Mapping reads to reference genome

High-quality reads in each sample were mapped to the reference genome provided by the customer. The analysis tool was Bowtie2 (http://bowtie-bio.sourceforge.net/bowtie2/index.shtml).

#### 2.6.2 rRNA contamination assessment

In this step, 10,000 randomly selected raw reads in each sample were aligned to the Rfam database (http://rfam.xfam.org/) with the blast method. Based on the annotation results, the percentage of rRNA in each sample was calculated, which was estimated as rRNA contamination. The analysis tool was Blast.

### 2.7 Quantitative PCR analysis

Total cellular RNA was extracted using TRIzol Reagent (Life Technologies, Carlsbad, CA, USA; 15596018) following the manufacturer’s instructions. A reverse transcription reaction was performed to produce complementary DNAs using a HiScript II Q RT Supermix for qPCR (+gDNA wiper) kit (Vazyme Biotech, Nanjing, China; R223). The complementary DNAs were subjected to quantitative real-time PCR (qRT-PCR) using an AceQ qPCR SYBR Green Master Mix Kit (Vazyme Biotech, Q111) and an ABI StepOne Plus Real-time PCR instrument (Applied Biosystems, Foster City, CA, USA). The relative quantification of gene expression was based on the threshold cycle (Ct), and rpoD was selected as the internal reference gene. Data were normalized to a control value of 1, and the relative expression levels of the target genes were calculated using the 2^−ΔΔCT^ approach (Doijad et al., 2023). The primers used for qRT-PCR are provided in **Supplementary Table S3**.

### 2.8 Fluorescence staining

The experimental isolates were maintained for 4 days with an induction dose of 1/16 MIC of IPM. This was followed by an IPM dose of 8× MIC. Then, residual cells were sampled 8 h after treatment, and resistant cells were sampled 24 h later. Isolates were cultured in a drug-free environment as controls. Cells in different states were fluorescently stained in parallel. An equal amount of bacterial suspension, at a concentration of approximately 2 McFarland, was transferred to an Eppendorf (EP) tube. After centrifugation, the medium was removed and washed with PBS. Each tube was then added with 50 μL of SYTO9/propidium iodide (PI) dye premix (Thermo Fisher, Waltham, MA, USA; L7012) and incubated for 15 min away from light (Ou et al., 2019; Alexander et al., 2020). After carefully removing the dye, the cells were transferred to a slide and observed under a confocal microscope (Olympus FV3000, 60× objective lens). The excitation light wavelengths selected were 480 nm for SYTO9 and 490 nm for PI.

### 2.9 Quantification of biofilms

Bacterial cells that underwent different phases were inoculated into a sterile 96-well plate. Each well contained 160 μL of Mueller–Hinton broth and 40 μL of bacterial suspension. The final concentration of bacterial cells was 5 × 10^5^ CFU/mL. The concentration of the antibiotic in each well was adjusted according to the different growth phases of the bacteria. All wells were seeded in triplicate and incubated at 35°C overnight. After incubation, the planktonic bacteria were removed by three times washing of plates using PBS, followed by desiccation for 20 min at room temperature.

Afterward, the biofilms were quantified by a crystal violet staining assay (Asadi et al., 2023). Crystal violet solution (Solarbio, China; C8470) at 0.1% (w/v) (200 μL) was added to each well and dyed at room temperature for 20 min. After that, the excess dye was removed, and the microplates were washed with 200 μL PBS. Finally, the residual stain was solubilized with 200 μL of 95% ethanol for 15 min. The quantification results were determined using a microtiter plate reader at OD590 nm (Thermo Scientific, Germany).

### 2.10 Transmission electron microscopy

Bacterial cells in different phases were harvested at a specified time, washed with PBS, and fixed with 2.5% (v/v) glutaraldehyde (EM grade; EMS, Electron Microscopy Sciences, Hatfield, PA, USA) at 4°C overnight. Afterward, the bacterial pellet was washed three times and post-fixed with osmium tetroxide (1% OsO_4_ in PBS) for 1 h. After three washing steps with water, additional en bloc contrast was achieved by incubating the samples in 2% (w/v) uranyl acetate in ddH_2_O overnight. Following three washing steps with water, bacterial pellet was dehydrated in a series of graded ethanol (50%, 70%, and 90% in ddH_2_O, 15 min each) and anhydrous acetone (90% in ddH_2_O, 15 min) and subsequently incubated in 100% anhydrous acetone (two times, 10 min each). The resulting sample was infiltrated with resin solution (SPI-Pon 812, SPI Supplies, West Chester, PA, USA), resin:acetone at 1:2 for 2 h, and then resin:acetone at 2:1 for 3 h, followed by pure resin incubation overnight and a final change of the pure resin the next day for another 3 h. A final embedment was performed in 100% resin at 37°C for 12 h, 45°C for 12 h, and 60°C for 48 h. Finally, samples were trimmed, and ultrathin sections (70 nm) were cut using an ultramicrotome equipped with a diamond knife (Leica EM UC7; Leica Microsystems, Wetzlar, Germany). The sections were collected on formvar–carbon-coated 300-mesh Cu grids and imaged on a FEI Tecnai F20 transmission electron microscope (FEI Company, Hillsboro, OR, USA) at an accelerating voltage of 200 keV. For the quantification of the thickness of the outer membrane, 25 bacterial cells were randomly selected from each group of samples, and the thickness of the outer membrane was measured as dispersed as possible at five locations around the bacterial cells.

### 2.11 Statistical analysis

Data were analyzed using the GraphPad Prism 9 software. Comparison between the two groups was conducted using Student’s t-test. Multiple group comparisons were performed using one-way ANOVA test. *p*-Values ≤0.05 were considered statistically significant.

## 3 Results

### 3.1 Unstable IPM-heteroresistant subpopulations in a clinical *E. coli* strain

We isolated a clinical *E. coli* strain, designated as ID1073, from a patient who contracted a hospital-acquired infection but without prior exposure to antibiotics. This ID1073 strain demonstrated resistance to multiple antibiotics, including levofloxacin, cefepime, and amikacin. Despite the antibiotic susceptibility tests suggesting that the strain was susceptible to IPM (group A), its heterogeneous subpopulation (group C) exhibited a MIC of 4 μg/mL, indicative of a highly resistant state to IPM (**Table 1**). PAPs showed the resistant subpopulations within the ID1073 strain capable of growth at IPM concentrations between 0.25 and 2 μg/mL, which is one to eight– times its MICs, thus confirming IPM heteroresistance (**Figure 1A**). Additionally, we discovered that these highly resistant subpopulations exhibited phenotypic plasticity and were independent of stable genetic inheritance.

**Table 1:** Antimicrobial susceptibility results of each group.

Antibiotic	Group A^a^	Interpretation	Group C^b^	Interpretation	Group B^c^	Interpretation	ATCC 25922	Expected
Amoxicillin-clavulanic acid	16	I	16	I	16	I	4	≤2-8
Cefuroxime	≥64	R	≥64	R	≥64	R	4	2-8
Ceftriaxone	≥64	R	≥64	R	≥64	R	≤0.25	≤0.25
Cefoperazone/Sulbactam	16	S	16	S	16	S	≤8	≤8
Cefepime	16	R	16	R	16	R	≤0.12	≤0.12
Imipenem	≤0.25 (0.25)	S	4(4)	R	≤0.25 (0.25)	S	≤0.25 (0.125)	≤0.25
Amikacin	≥64	R	≥64	R	≥64	R	≤2	≤2-4
Levofloxacin	≥8	R	≥8	R	≥8	R	≤0.12	≤0.12
Tigecycline	≤0.5	S	≤0.5	S	≤0.5	S	≤0.5	≤0.5
Trimethoprim/Sulfamethoxazole	≥320	R	≥320	R	≥320	R	≤20	≤20

a Group A:1073 original replicated strain, including A1, A2, A3 isolates.

b Group C: an IPM highly resistant subclone, including C1, C2 replicated isolates.

c Group B: reversible isolates derived from group C, including B1, B2, B3 replicated isolates.

The MIC values were measured by the instrument method, and the values in ( ) are measured by double dilution method; MIC (μg/mL).

In the serial passage experiment (**Figure 1B**), we observed that highly resistant subpopulations could grow sustainably in Mueller–Hinton broth supplemented with the same selection pressure (IPM, 2 μg/mL) as the plate from which the clone originated. These subclones exhibited a stable resistant phenotype, with at least >50% frequency of the cells from each passage exhibiting IPM resistance (**Figures 1C–E**). However, the purely and highly resistant clones were cultured under IPM-free conditions (**Figure 1B**), and none were able to maintain a stable resistant phenotype. Instead, they only demonstrated a temporary heteroresistant phenotype. Furthermore, in subsequent generations, we observed a gradual reduction in the frequency of heteroresistant colonies. Ultimately, by the fifth generation, all colonies had reverted to a sensitive and homogeneous state (**Figures 1C–E**).

### 3.2 Transcriptomic signatures of highly IPM-resistant subpopulations

To gain insights into the underlying mechanisms, experimental isolates were selected from the above serial passage for further sequencing study (**Figure 1B**, **Tables 1**, **2**). Group A displayed an IPM-heteroresistant phenotype. Group C was a pure subclone from the first PAP assay that was highly IPM-resistant. Group B was susceptible and homogeneous toward IPM, and its lineage was derived from group C (**Table 2**). Antibiotic phenotypes in each group are listed in **Table 1**.

**Table 2:** List and information of isolates for sequencing analysis.

Group	Isolate name	Isolate description	Phenotype	Phenotype description
A	A1073_1	1073 original replicated isolates	Heteroresistance	Contained subpopulations (approximately 0.05-0.1‰ of the cells) with high levels of resistance
	A1073_2			
	A1073_3			
C	C1073_1	Replicated isolates; from initial PAPs	Highly resistance	Pure highly resistance with 8 fold IPM MIC value
	C1073_2			
B	B1073_1	Replicated isolates; from serial passage ⑤	Susceptibility and homogeneity	Contained subpopulations range of IPM MIC ≤ 2 fold value
	B1073_2			
	B1073_3			

**Figure 2:**
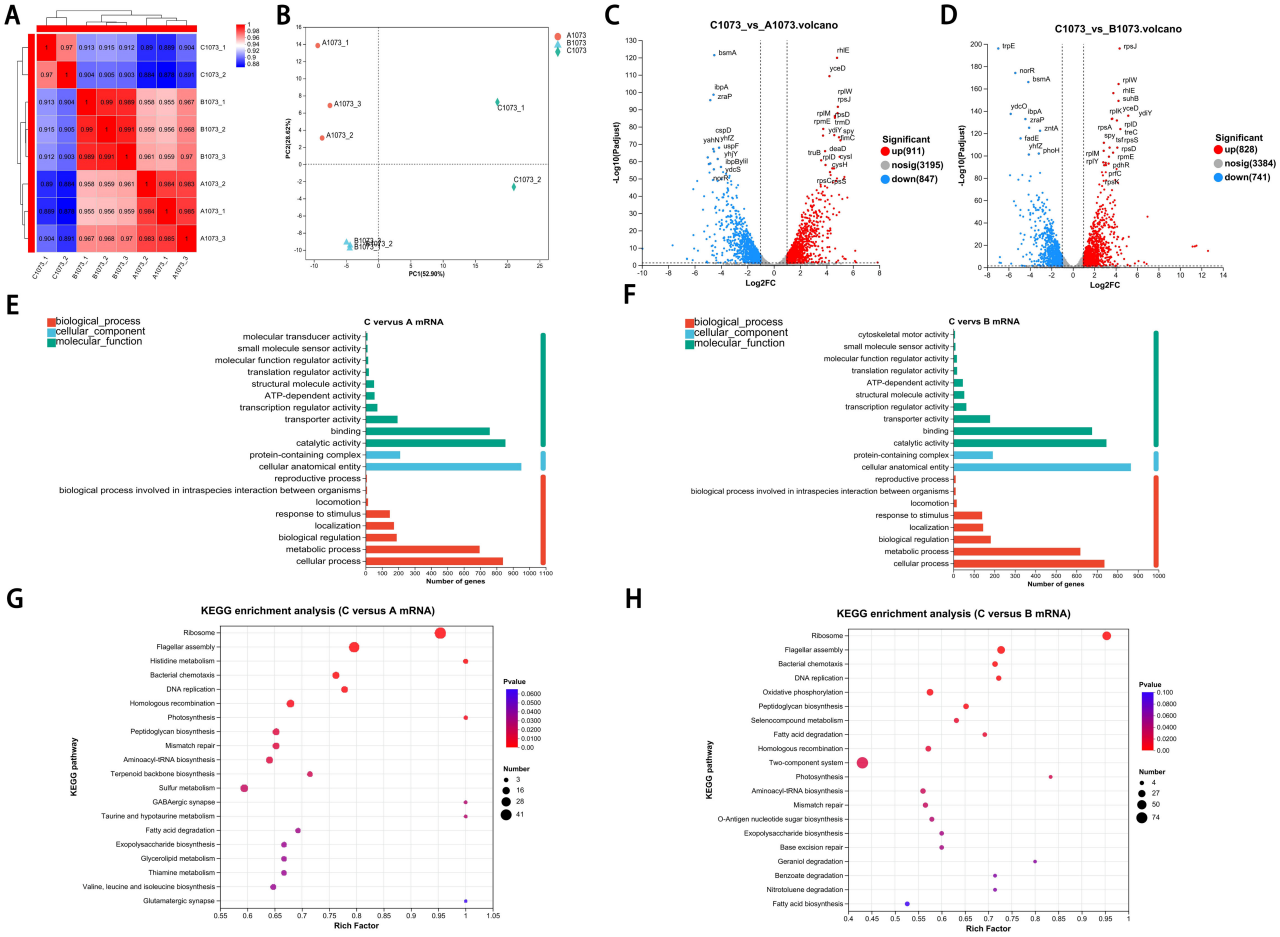
Transcriptomic signatures of highly resistance subpopulation.**(A)** Correlation coefficients among three groups. **(B)** PCA showed the relative independent clusters among the three groups. **(C, D)** Volcano plots displaying DEGs in two comparative groups. **(E, F)** GO analysis of DEGs in different gene sets. **(G, H)** KEGG pathway enrichment analysis of the annotated DEGs in two comparative groups.

Although the three groups exhibited distinct IPM phenotypes, through whole-genome sequencing, we confirmed that no genetic mutations were detected in clinically recognized IPM-resistant gene loci (KPC, NDM, OXA, VIM, IMP, IMI, SME, CTX-M, SHV, and TEM) (Nordmann and Poirel, 2019). However, transcriptome sequencing analysis showed that, despite the high correlation coefficients observed among the eight samples with each other (**Figure 2A**), principal component analysis (PCA) still enabled us to identify three independent clusters corresponding to the three distinct phenotypes, separately. Notably, in PC1, there was a clear separation of group C from groups A and B, highlighting the presence of more unique transcriptomic features in the highly IPM-resistant subpopulations (**Figure 2B**).

Volcano plots show that genes in group C had a considerable number of differentially expressed genes (DEGs) compared with groups A and B (**Figures 2C, D**). To further obtain a biological functional insight into these DEGs, GO annotations and KEGG pathway analyses were performed (**Figures 2E–H**). We found that, whether compared to those in group A or B, the DEGs in group C shared similar GO annotation characteristics, all summarized in the three main categories. In the biological process category, the GO terms that were significantly enriched included “cellular process” and “metabolic process”. In the cellular component category, “protein-containing complex” and “cellular anatomical entity” were significantly enriched. In the molecular function category, “catalytic activity” and “binding” were significantly enriched (**Figures 2E, F**). From the KEGG analysis, we also found that these DEGs had high similarity in pathway enrichment, regardless of whether compared to group A or B (**Figures 2G, H**). Overall, group C represented the purely and highly IPM-resistant phenotype with specific transcriptional profiles.

### 3.3 Reversible regulatory networks in unstable IPM-resistant populations

**Figure 3:**
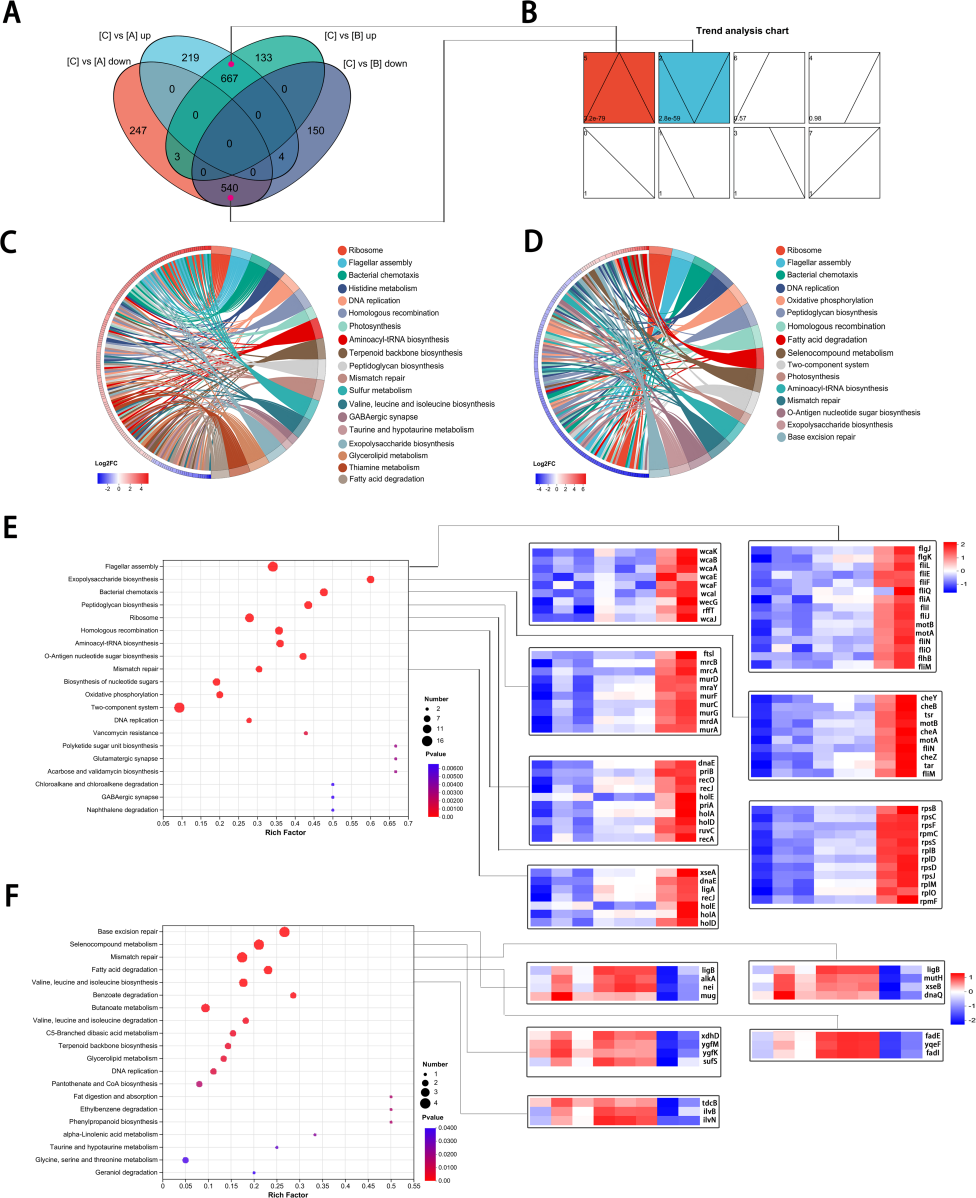
Reversible gene expression panels involved in unstable IPM-resistant populations. **(A)** Venn diagram showing shared and DEGs in four comparative groups. **(A, B)** The overlapped genes in cyan and purple, respectively, correspond to the 5 and 2 gene expression panels. **(B)** Classification of the DEGs based on their expression pattern. The top left corner: panel number; the lower left corner: *P* value; box in color showed statistical significance. **(C, D)** Chord diagram showing the distribution and expression status of DEGs in the relevant pathways. **(C)** C versus A DEGs set. **(D)** B versus C DEGs set. **(E, F)** KEGG pathway enrichment analysis of the DEGs (left) and heat map of representative genes (right). **(E)** KEGG pathway enrichment analysis for 110 genes with panel 5. **(F)** KEGG pathway enrichment analysis for 24 genes with panel 2.

To analyze the expression patterns of DEGs involved in group C, we further divided DEGs into four comparative gene sets. The Venn diagram shows that compared with those in groups A and B, there were 667 upregulated and 540 downregulated genes in group C. Furthermore, these genes account for the majority (**Figure 3A**). The trend chart further depicts that there were eight kinds of gene expression patterns among these DEGs. Nevertheless, only patterns 5 and 2 were statistically significant (graph in color), corresponding to the patterns of DEG expression that were only upregulated or downregulated in group C, respectively (**Figures 3A, B**). To further explore these key DEGs involved in dynamic change related to the unstable IPM-resistant phenotype, we also compared the distribution and expression status of DEGs in the relevant pathways. In the C versus A DEG set, which corresponded to the highly resistant versus heteroresistant phenotype, the chord diagram shows that genes in most of the transcriptomic pathways had significantly upregulated expression. Moreover, only a limited number of pathways, such as the fatty acid degradation pathway, displayed downregulation (**Figure 3C**). In the B versus C DEG set, which corresponded to the susceptibility versus highly resistant phenotype, these DEGs within the same pathways exhibited obvious opposite regulations (**Figure 3D**). It indicated that specific reversible regulations of transcriptomic pathways were also present in the phenotype switch between high resistance and susceptibility.

Among DEGs in pattern 5, we identified 110 genes that were assigned to the associated top 20 pathways, and in pattern 2, there were 24 genes (**Supplementary Tables S1**, **S2**). Among these genes, in addition to a high proportion of enrichment still in the ribosome, flagellar assembly pathways, they were also involved in the peptidoglycan biosynthesis, exopolysaccharide biosynthesis, homologous recombination, and mismatch repair pathways (**Figures 3E, F**), indicating that the highly resistant subpopulations in group C share characteristics of bacterial survival under IPM treatment stress.

**Figure 4:**
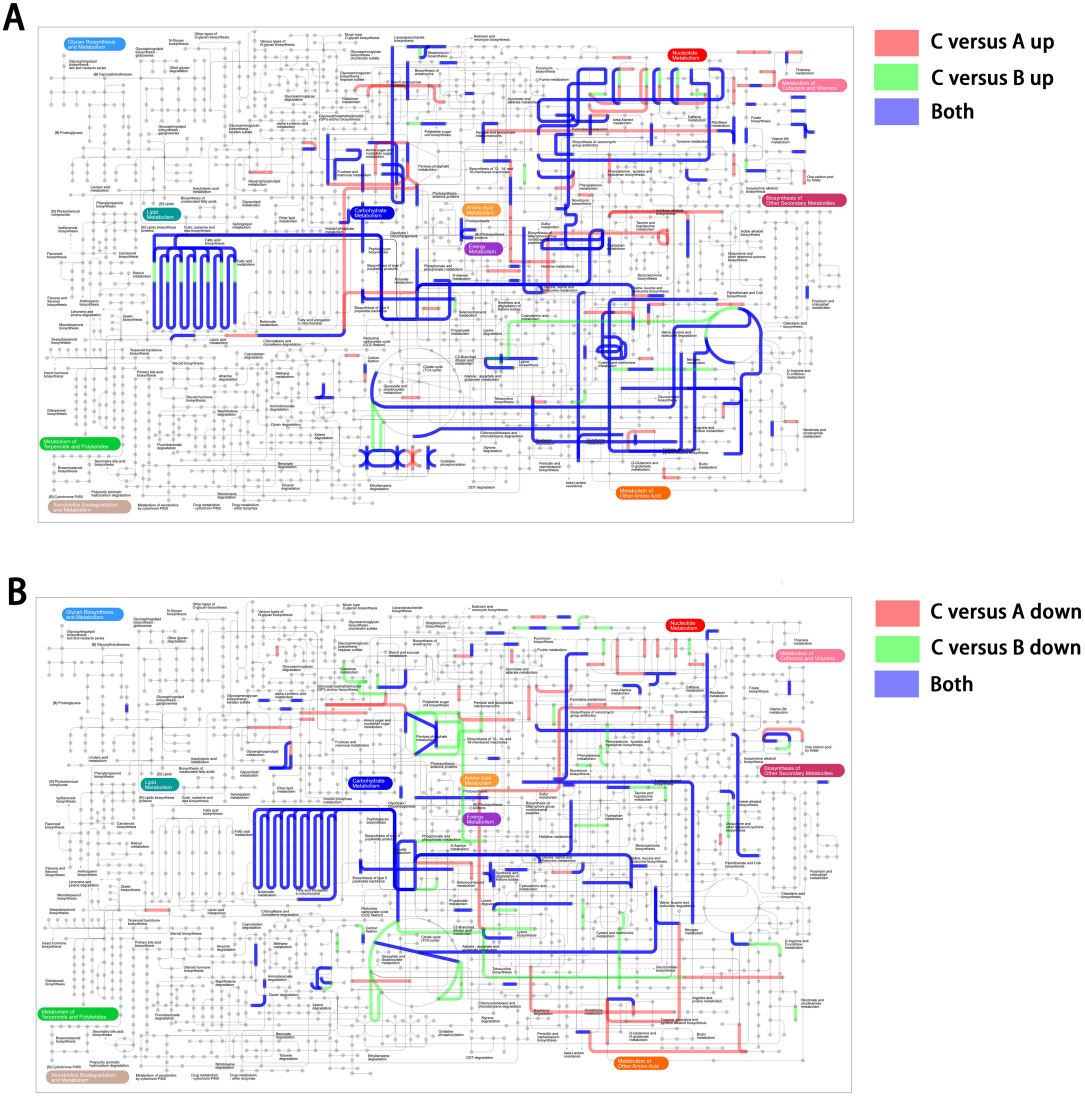
The iPath showing an overview metabolic and regulatory network involved in unstable IPM-resistant phenotype. **(A)** DEGs belonging to pattern 5. **(B)** DEGs belonging to pattern 2.

To better understand the interaction of the DEGs in the transcription network, we mapped an overview of pathways associated with the DEGs using iPath (Letunic et al., 2008). The circuit map shows that DEGs belonging to pattern 5 (**Figure 4A**) or pattern 2 (**Figure 4B**) were enriched in most pathways (blue lines), such as lipid metabolism, carbohydrate metabolism, nucleotide metabolism, energy metabolism, and amino acid metabolism. Additionally, it is worth noting that these pathways associated with genes in patterns 5 and 2 did not show obvious overlaps, suggesting that DEGs with opposite expression trends may collectively play a complementary role, contributing to the regulation of the reversible IPM-resistant phenotype.

### 3.4 Low level of IPM regenerated heteroresistance with similar transcriptional features

To demonstrate the reproducibility of the unstable IPM-heteroresistant phenotype and its regulatory mechanisms, we selected group B as experimental isolates (**Tables 1**, **2**; B1–B3 isolates) and applied IPM as a trigger to induce the reemergence of heteroresistance (Eisenreich et al., 2022). To rule out potential resistance mechanisms caused by selective mutations due to high-dose IPM (Couce and Blázquez, 2009; Baquero et al., 2021; Helekal et al., 2023), low-level IPM induction would be a suitable choice (Westhoff et al., 2017).

Prior to assessing the reproducibility of the IPM-heteroresistant assays, we rigorously validated the experimental homogeneous isolates to ensure that they only exhibited limited clonal survival at 1× MIC of imipenem. Notably, there were already no detectable heteroresistant subpopulations that were observed at 2× MIC or higher concentrations of imipenem. In such a scenario, these validated clones were thus utilized in subsequent experiments to investigate the regeneration of heteroresistant populations under sub-MIC pressure.

**Figure 5:**
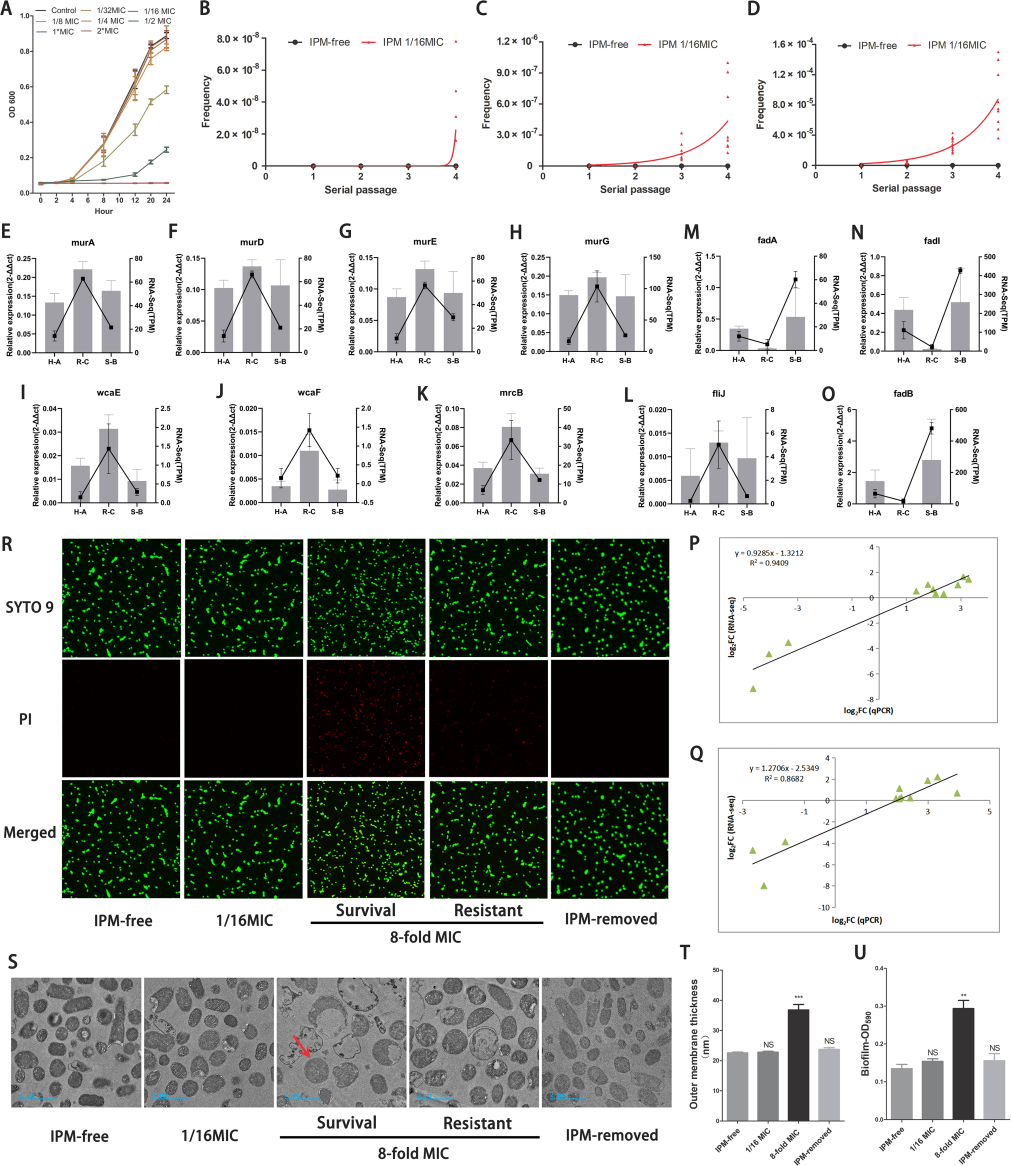
Similar transcriptional and biological features verified by PCR and functional studies. **(A)** The growth rates of isolates in group B under different IPM levels. **(B-D)** Frequencies of IPM resistant cells among B1-B3 isolates in each passage. IPM-free: isolates without IPM as reference; IPM 1/16MIC: isolates with the IPM-induced level (0.0156µg/ml). The X-axis represents the induced serial passage. Each point on the Y-axis represents the frequency of IPM-resistant cells in each isolate (B1-B3 isolates was replicated independently three times). Plates from PAP assay with different IPM concentration: **(B)** 8×MIC, **(C)** 4×MIC, **(D)** 2×MIC. **(E-O)** Verifcation of DEGs using qRT-PCR. Note: The relative gene expression level was calculated by 2^-ΔΔCT^. H-A: regenerated heteroresistance vs group A; R-C: regenerated resistance vs group C; S-B: regenerated IPM-sensitive revertant vs group B **(P, Q)** Linear regression of qRT-PCR and RNA-Seq data that are expressed as a log2 fold change. **(P)** C versus A group; **(Q)** C versus B group. **(R)** Fluorescence staining images of bacterial cells illustrating the dynamic integrity states of cell outer membrane following IPM treatment. Intact or disrupted cell structure was visualized by SYTO 9 staining (green), disrupted cell structure was stained by PI (red). **(R, S)** [Survial]: survival populations; [Resistant]: highly resistant populations. **(S)** The micrographs from TEM unveiled the dynamic morphology changes of the bacterial ultrastructure. Compared to untreated controls (IPM-free), subpopulations pre-exposed to sub-inhibitory IPM (1/16×MIC) exhibited no detectable morphological alterations. In stark contrast, IPM-resistant subpopulations surviving 8×MIC IPM concentrations exhibited intact outer membrane (red arrow), while most IPM-sensitive populations are experiencing rupture of their outer membrane. Surviving cells continued to grow under the lethal IPM concentrations. Notably, when under IPM-removed conditions, these resistant cells could still return to their original morphology. **(T)** Quantification of the thickness of the outer membrane. **(U)** Quantifcation of biofilms of bacterial populations in different conditions. **(T, U)** IPM-free: B1-B3 isolates culture without IPM; IPM-removed: B1-B3 isolates underwent induction and treatment with 1/16 and 8×MIC of IPM, followed by recovery in IPM-free environment. **: P<0.01, ***: P<0.001, NS: no statistical significance.

**Figure 5A** clearly illustrates that the homogeneous and IPM-sensitive bacterial population maintained a relatively unaffected growth curve under an IPM concentration of 1/16 MIC, compared to a drug-free condition. Utilizing this IPM dose, we conducted a serial passage induction experiment. Each isolate underwent three replicated cultures, separately. The results indicated that during the induction period, all experimental isolates met the criteria for heteroresistance (**Figures 5B–D**). Some subpopulations even exhibited resistance to 2 μg/mL (8× MIC) IPM concentrations within 40 generations (**Figure 5B**).

To determine whether reproducing heteroresistant populations may bear a resemblance to the transcriptional signature of the original ID1073 clinical strain, related genes were chosen for correlation analysis by qRT-PCR validation. As expected, although the relative fold change of gene expression was not exactly consistent between the sequencing and qRT-PCR data, the expression patterns of these genes were very similar to those of the Fragments Per Kilobase of exon model per Million mapped fragments (FPKM) values from sequencing. These genes involving the peptidoglycan biosynthesis, exopolysaccharide biosynthesis, and other pathways displayed increased expression status in highly resistant populations similar to pattern 5 (**Figures 5E–L**). Moreover, other genes such as fadA, fadB, and fadI, which are involved in the fatty acid degradation pathway, exhibited decreased expression status in highly resistant populations similar to pattern 2 (**Figures 5M–O**). The correlation coefficient in the C versus A group (**Figure 5P**) and the C versus B group (**Figure 5Q**) further confirmed the similar adaptive transcriptional signature in these IPM-induced, reproduced heteroresistant populations.

### 3.5 Characteristic cellular structures in heteroresistant subpopulations

Using fluorescence staining and transmission electron microscopy (TEM), we monitored the temporal alteration of cell structure in heteroresistant subpopulations across various stages, including the IPM-sensitive, survival, and resistant phases, as well as the revertant phase. At a concentration of 1/16 MIC of IPM, co-labeling SYTO 9-PI and TEM revealed that the bacterial cell outer membranes remained relatively intact (**Figures 5R, S**). An 8× IPM MIC induced cell wall breakage and leakage of cellular contents in most of the bacterial cells. However, the surviving bacterial cells maintained the integrity of their cellular structure (**Figures 5R, S**) and afterward exhibited resistance to IPM by strengthening their outer cellular structure, such as thickening of the outer membrane, which is the formation of biofilms (**Figures 5T, U**). In IPM-free conditions, these heteroresistant bacterial cells recover their changes in cellular and intercellular structures, returning to their normal morphological state (**Figures 5R–U**).

## 4 Discussion

It has recently been recognized that bacterial subpopulations are not static and separate entities but rather interconnected and dynamically changing (Mukherjee and Bassler, 2019). Our study revealed a dynamic shift in course among unstable IPM-resistant states in *E. coli* populations, exhibiting adaptive responses across varying environments, which could steer them to potentially divergent evolutionary paths.

When the whole bacterial population was in a state of heteroresistance, the highly resistant subpopulations could undergo strong selection of 8× IPM MIC compared with the dominant populations with a sensitive phenotype and become residual bacteria. These highly resistant bacterial populations displayed a distinct transcriptional signature. The majority of enriched pathways showed significant upregulation in expression. Our subsequent IPM induction studies further verified that the similar gene expression patterns related to bacterial physiology and structure, such as those involved in cell outer membrane synthesis (mrcB, murA, murD, murF, and murG) and biofilm formation (wcaE and wcaF), were enhanced in highly resistant subpopulations. Also of note, a subset of pathways and gene expressions were observed to be downregulated, such as those involved in fatty acid degradation (fadA, fadB, and fadI). Recent studies have hinted that the synthesis and degradation of fatty acids may lead to changes in the composition of phospholipids, which in turn also alter the bacterial–physiological structure, such as cell membranes, and thus affect antibiotic resistance (Lambert C et al., 2022).

When these highly resistant bacterial cells were subsequently under an antibiotic-removed environment, they efficiently lost their highly resistant phenotype, accompanied by reversible changes in transcriptome expression patterns. As demonstrated in this study, the ways that strains evolve by gene-regulation means are more susceptible to external environmental perturbations, offering a plausible explanation for the rapid acquisition/loss of heteroresistance in bacterial strains.

The origins of heteroresistance have long been a subject of debate, with controversy surrounding whether it arises inherently and spontaneously or through other means (Nicoloff et al., 2019; Kapel et al., 2022; Sun L et al., 2024). In our study, we isolated the ID1073 strain from a patient who had not received any prior antibiotic treatment yet exhibited a heteroresistant phenotype. These heteroresistant subpopulations appeared to be present inherently, without having been subjected to significant therapeutic selection pressure or other identifiable triggers. However, we found contradictory facts that these heteroresistant subpopulations hardly maintained a highly resistant state under normal IPM-free conditions. Furthermore, once highly resistant populations reverted to their sensitive state, they were not able to survive subsequent IPM treatment. These observations suggest that the maintenance of heteroresistant subpopulations in our *E. coli* strain is not dependent on simple genetic predisposition or does not exist spontaneously. Instead, it suggests that additional and continuous environmental stimuli are still required. Combined with the clinical background of nosocomial infection, we have to consider that heteroresistance is still related to environmental factors. Nevertheless, different levels of environmental stimulation may influence strains to react by distinct mechanisms (Hughes and Andersson, 2012; Baquero et al., 2021; Roemhild et al., 2022). To rule out the genetic predisposition induced by antibiotic pressure, we employed low-level IPM exposure to induce the reemergence of heteroresistance. The result showed that highly resistant subpopulations can still be regenerated without a trade-off in fitness, displaying a similar heteroresistant phenotype and transcriptional profile as the original clinical strain did. Combined with the clinical context, it is conceivable that the extremely low antibiotic levels may be present in many clinical settings contaminated by antibiotics (Liu et al., 2021). This is the most likely explanation why many hospital-acquired infection cases that have not been previously treated with antibiotics have pre-existing unstable heteroresistance.

Previous studies have also confirmed that low-level IPM antibiotic exposure can select for resistant bacteria (Hughes and Andersson, 2012; Liu et al., 2021). However, they still demonstrated from the perspective of genetic mechanism and only after long-term serial passage experiment (600–900 generations), making the highly fully resistant phenotype possible by the step-wise accumulation of resistance mutations with individually smaller effects (Westhoff et al., 2017; Wistrand-Yuen et al., 2018). In our study, the spans of the antibiotic induction experiment were very short, especially in an experiment with 30 to 50 serial passages. It appears improbable that bacterial strains can establish fixed evolutionary paths between sensitive and resistant states through potent genetic mechanisms (Good et al., 2017; Wistrand-Yuen et al., 2018). The extremely low-level IPM should not be perceived merely as an environmental stress. Rather, it should be considered more akin to signaling molecules, rendering *E. coli* strains more receptive to environmental shifts and preparing them for potential adverse conditions that could arise.

In addition, it should be noted that transcriptional regulatory mechanisms can rapidly alter the physiological structures of bacterial cells, such as extracellular membranes and biofilms. Admittedly, these structural changes may also potentially alter the efficacy of other antibiotics. Further studies on changes in secondary drug susceptibility in heterogeneous resistant populations will be useful for the clinical prediction of effective antimicrobial strategies.

Overall, our study reveals the dynamic changes in phenotype and key gene regulation of bacterial heteroresistant populations in response to environmental variations. These non-genetic mechanisms enable bacterial strains to acquire environmental adaptability more rapidly. Moreover, preventing hospital-acquired infections focuses on not only the elimination of residual bacteria but also the removal of residual antibiotics in clinical settings.

## Data Availability

The original contributions presented in the study are publicly available. This data can be found here: https://www.ncbi.nlm.nih.gov/sra under accession number PRJNA1145105.
