# Supplementary material for: Heterogeneous subpopulations in Escherichia coli strains acquire adaptive resistance to imipenem treatment through rapid transcriptional regulation
Source: Front Cell Infect Microbiol. 2025 May 30;15:1563316. doi: 10.3389/fcimb.2025.1563316 (PMC12163615; doi:10.3389/fcimb.2025.1563316)
Supplement: Supplementary file 1 [file Table1.docx]

**Supporting Information**

**Table S1.** The gene name and descriptions for 110 genes in panel 5.

| Gene ID | Gene Name |
| --- | --- |
| K758_RS18885 | wecG |
| K758_RS11520 | glsB |
| K758_RS23420 | atpD |
| K758_RS20300 | ispC |
| K758_RS12410 | argS |
| K758_RS06890 | valS |
| K758_RS09720 | glnA |
| K758_RS19710 | thiL |
| K758_RS13585 | tyrS |
| K758_RS18895 | rffT |
| K758_RS04980 | alaS |
| K758_RS19680 | thiI |
| K758_RS15050 | ligA |
| K758_RS09945 | wcaK |
| K758_RS21305 | ispH |
| K758_RS21025 | ftsI |
| K758_RS10415 | dhaL |
| K758_RS21010 | mraY |
| K758_RS07860 | holA |
| K758_RS07885 | mrdA |
| K758_RS03215 | murA |
| K758_RS10645 | adhE |
| K758_RS09395 | priA |
| K758_RS21005 | murD |
| K758_RS20190 | mrcB |
| K758_RS14405 | recO |
| K758_RS16780 | wcaA |
| K758_RS03975 | yiaY |
| K758_RS01740 | lysS |
| K758_RS18980 | ilvC |
| K758_RS20990 | murC |
| K758_RS20355 | dnaE |
| K758_RS20995 | murG |
| K758_RS10460 | ispE |
| K758_RS12385 | cheY |
| K758_RS21595 | holD |
| K758_RS12475 | ruvC |
| K758_RS16840 | wcaJ |
| K758_RS04575 | plsX |
| K758_RS13205 | pheS |
| K758_RS12365 | tar |
| K758_RS20880 | tsr |
| K758_RS12390 | cheZ |
| K758_RS23425 | atpG |
| K758_RS21015 | murF |
| K758_RS12345 | motA |
| K758_RS16800 | wcaE |
| K758_RS12085 | fliM |
| K758_RS04610 | flgK |
| K758_RS16805 | wcaF |
| K758_RS12090 | fliL |
| K758_RS16785 | wcaB |
| K758_RS12075 | fliO |
| K758_RS16825 | wcaI |
| K758_RS06595 | priB |
| K758_RS12065 | fliQ |
| K758_RS12380 | cheB |
| K758_RS12105 | fliI |
| K758_RS03045 | rplM |
| K758_RS12355 | cheA |
| K758_RS04580 | rpmF |
| K758_RS20280 | rpsB |
| K758_RS18225 | rplD |
| K758_RS12100 | fliJ |
| K758_RS18135 | rplO |
| K758_RS04615 | flgJ |
| K758_RS12200 | fliA |
| K758_RS12080 | fliN |
| K758_RS15000 | cysA |
| K758_RS05300 | cysH |
| K758_RS18215 | rplB |
| K758_RS05275 | cysC |
| K758_RS18210 | rpsS |
| K758_RS18110 | rpsD |
| K758_RS18235 | rpsJ |
| K758_RS12350 | motB |
| K758_RS07980 | citX |
| K758_RS16810 | gmd |
| K758_RS16765 | wza |
| K758_RS07590 | kdpA |
| K758_RS18200 | rpsC |
| K758_RS18190 | rpmC |
| K758_RS06590 | rpsF |
| K758_RS12395 | flhB |
| K758_RS16815 | fcl |
| K758_RS12125 | fliE |
| K758_RS10005 | cpsG |
| K758_RS12120 | fliF |
| K758_RS18425 | fdnH |
| K758_RS0124255 | fdnG |
| K758_RS16830 | cpsB |
| K758_RS22415 | mrcA |
| K758_RS06735 | ppa |
| K758_RS13610 | nth |
| K758_RS12585 | holE |
| K758_RS15640 | nuoI |
| K758_RS18910 | rffC |
| K758_RS17840 | frdA |
| K758_RS20645 | proS |
| K758_RS15115 | gltX |
| K758_RS15620 | nuoE |
| K758_RS15635 | nuoH |
| K758_RS17835 | frdB |
| K758_RS04995 | recA |
| K758_RS16520 | metG |
| K758_RS18915 | rfbA |
| K758_RS18905 | rffA |
| K758_RS18920 | rffG |
| K758_RS14670 | xseA |
| K758_RS01730 | recJ |

**Table S2.** The gene name and descriptions for 24 genes in panel 2.

| Gene ID | Gene Name |
| --- | --- |
| K758_RS09145 | tdcB |
| K758_RS17180 | fadE |
| K758_RS15345 | fadI |
| K758_RS01945 | mutH |
| K758_RS09320 | gldA |
| K758_RS06235 | dnaQ |
| K758_RS01880 | yqeF |
| K758_RS09160 | garK |
| K758_RS23140 | ilvN |
| K758_RS22130 | ggt |
| K758_RS15495 | ubiX |
| K758_RS22885 | ligB |
| K758_RS19685 | xseB |
| K758_RS23145 | ilvB |
| K758_RS08915 | mug |
| K758_RS13375 | sufS |
| K758_RS16735 | alkA |
| K758_RS07540 | nei |
| K758_RS02330 | cydX |
| K758_RS03755 | dppA |
| K758_RS01800 | ygfK |
| K758_RS01790 | ygfM |
| K758_RS17220 | zraP |
| K758_RS01785 | xdhD |

**Table S3.** The primer sequences used for qRT-PCR.

| Gene | Primer sequence | The number of bases |
| --- | --- | --- |
| fadA-U | CCCGCTCAAACACCGTCGCAATA | 23 |
| fadA-D | CCTTTGCCGCGCAGATCCT | 19 |
| fadB-U | CAGCACCGAGCATACGTGGCATA | 23 |
| fadB-D | TGCACTTTGCCAATAGCGTGTTT | 23 |
| fadI-U | CTCATTGTCAAACCGGCACGTTC | 23 |
| fadI-D | CGTCCGGCGTTTGATCGTAA | 20 |
| fliJ-U | TGAAATGCGTCGCGGATGT | 19 |
| fliJ-D | TGCTGGCGATGCTGAGTAATGGC | 23 |
| murG-U | TTCCCAGGGCGCACGCATTCT | 21 |
| murG-D | CGACGACATCCGCCCACGCATAC | 23 |
| murE-U | GCTGGTGGATCAGGGCGCGACT | 22 |
| murE-D | CAGCGGCGGCCCACTTCATCGTC | 23 |
| mrcB-U | GCTGCTCACCGTTTGGCGAAGAA | 23 |
| mrcB-D | TGACCCGTCCTGGCGAGTTTACC | 23 |
| murD-U | ACTGGTCTGCCGCATCGCTTTGA | 23 |
| murD-D | CGCGCCGTCACGACCGAAAC | 20 |
| murA-U | CCGTCGTCCCTTCCGCAATACA | 22 |
| murA-D | GTGGCAGAAGGGACCGGATTCAT | 23 |
| wcaF-U | CCACATTTGCCGGAAGCGATTT | 22 |
| wcaF-D | ACCATTGGCGCACATTCGGTGAT | 23 |
| wcaE-U | GTTCATGGCGTCGTAGATACCGT | 23 |
| wcaE-D | TCACTGTCGCGTTTCGTAACCTC | 23 |
